# Obinutuzumab in Combination with Alternative Chlorambucil Schedules in Front-Line Treatment of Chronic Lymphocytic Leukemia: A Study by KroHem, the Croatian Cooperative Group for Hematologic Diseases

**DOI:** 10.3390/biomedicines12122902

**Published:** 2024-12-20

**Authors:** Igor Aurer, Ozren Jakšić, Sandra Bašić-Kinda, Karla Mišura-Jakobac, Jasminka Sinčić-Petričević, Sabina Novaković-Coha, Davor Galušić, Hrvoje Holik, Toni Valković, Dubravka Županić-Krmek, Ida Hude-Dragičević, Vibor Milunović, Vlatko Pejša

**Affiliations:** 1 Division of Hematology, Department of Internal Medicine, University Hospital Centre Zagreb and Medical School, University of Zagreb, 10000 Zagreb, Croatia; 2 Division of Hematology, Department of Internal Medicine, University Hospital Dubrava and Medical School, University of Zagreb, 10000 Zagreb, Croatia; ojaksic@kbd.hr (O.J.); vlatko.pejsa@kbd.hr (V.P.); 3 Division of Hematology, Department of Internal Medicine, University Hospital Centre Zagreb, 10000 Zagreb, Croatia; sandra.kinda@gmail.com (S.B.-K.); ida.hude@gmail.com (I.H.-D.); 4 Division of Hematology, Department of Internal Medicine, University Hospital Merkur, 10000 Zagreb, Croatia; karlamisura@gmail.com (K.M.-J.); v_milunov@net.hr (V.M.); 5 Division of Hematology, Department of Internal Medicine, University Hospital Centre Osijek, 31000 Osijek, Croatia; sincicpetricevic@yahoo.com; 6 Division of Hematology, Department of Internal Medicine, University Hospital Centre Sisters of Mercy, 10000 Zagreb, Croatia; snovakovic89@gmail.com; 7 Division of Hematology, Department of Internal Medicine, Division of Haematology, University Hospital Centre Split, 21000 Split, Croatia; davorgalusic@net.hr; 8 Hematology-Oncology Unit, Internal Medicine Service, General Hospital Dr. Josip Benčević, 35000 Slavonski Brod, Croatia; hholik@gmail.com; 9 Division of Hematology, Department of Internal Medicine, University Hospital Centre Rijeka and Medical School, University of Rijeka, 51000 Rijeka, Croatia; toni.valkovic@medri.uniri.hr; 10 Division of Hematology, Department of Internal Medicine, University Hospital Holy Spirit, 10000 Zagreb, Croatia; zupanicd@gmail.com

**Keywords:** chronic lymphocytic leukemia, obinutuzumab, chlorambucil

## Abstract

**Background/Objectives***:* Obinutuzumab was approved for front-line treatment of chronic lymphocytic leukemia in combination with chlorambucil pulses administered every 2 wks. Alternative schedules of chlorambucil enable the administration of higher total chlorambucil doses, and have better antileukemia activity. So far, evidence on the feasibility of combining obinutuzumab with alternative chlorambucil schedules is lacking. We performed this retrospective analysis to analyze real life outcomes in chronic lymphocytic leukemia patients receiving a combination of obinutuzumab with different chlorambucil schedules. **Methods:** This was a retrospective survey performed in order to analyze the feasibility and efficacy of different obinutuzumab and chlorambucil combinations in a real-life setting. Patients receiving this combination as a front-line therapy for chronic lymphocytic leukemia in participating centers, outside of clinical trials, in 2017 and 2018 were included. **Results:** Seventy-three patients fulfilling entry criteria were identified. Their median age was 76 years, and ranged from 58 to 90 years. The median follow up time was 59 months. The response rate was 89%, with a median progression-free survival time of 27 months, and an overall survival time of 49 months. Chlorambucil was administered as planned in 15 of the 22 (79%) patients treated with chlorambucil pulses every 2 weeks; in 15 of the 42 (34%) patients treated with 7-day courses of chlorambucil administered every 4 weeks; and in 0 of the 10 patients treated with a continuous high dose of chlorambucil (*p* = 0.002). Changes in treatment schedules were made due to side effects. The progression-free and overall survival rates were similar between the three groups. **Conclusions:** The combinations of obinutuzumab with more intensive chlorambucil schedules are less feasible, preventing the administration of the intended higher total dose of chlorambucil, and do not improve outcomes in comparison to chlorambucil pulses administered every 2 weeks.

## 1. Introduction

Obinutuzumab is a glycoengineered monoclonal antibody directed against CD20, an antigen expressed on most mature B lymphocytic cell types and mature B cell malignancies, including chronic lymphocytic leukemia. Obinutuzumab has been purposefully designed to have superior antibody-dependent cellular cytotoxicity, but not complement-dependent cytotoxicity, in comparison to rituximab [1,2]. The large prospective, international, randomized, phase III CLL11 study was a 3 arm study comparing the combination of obinutuzumab and chlorambucil with the combination of rituximab and chlorambucil and chlorambucil monotherapy in previously untreated unfit patients with chronic lymphocytic leukemia that were in need of therapy [3]. Being unfit was defined by either having a geriatric cumulative index rating scale score between 7 and 20, or creatinine clearance between 30 and 69 mL/min. The study enrolled 781 patients; 333 received the combination of obinutuzumab and chlorambucil, 330 received the combination of rituximab and chlorambucil, and 118 received chlorambucil monotherapy. Enrollment in the chemotherapy-only arm was stopped earlier because of inferior response and progression-free and overall survival rates, in comparison to the two immunochemotherapy arms. The response rate to the combination of obinutuzumab and chlorambucil was 78.4%, in comparison to 65.1% to the combination of rituximab and chlorambucil (*p* < 0.001). The median progression-free survival was 27 months in the former group and 15 months in the latter group (*p* < 0.001), resulting in a 61% reduced risk of progression or death (95% confidence interval 69% to 51%). After a median observation time of over 5 years, the difference in overall survival also became statistically significant (*p* = 0.0196; hazard ratio 0.68, 95% confidence interval 0.49–0.94), with overall survival rates at 5 years of 66% and 53%, respectively [4]. Based on this study, the combination of obinutuzumab and chlorambucil was approved for front-line treatment of chronic lymphocytic leukemia in unfit and elderly patients.

In the seminal CLL11 study, chlorambucil was administered at a dose of 0.5 mg/kg every 2 weeks, on days 1 and 15 of each 4-week cycle for 6 cycles. This results in a total dose of 6 mg/kg or approximately 250 mg/m^2^ of chlorambucil. Alternative chlorambucil schedules, either continuous administration at doses of 10 to 20 mg daily until response or toxicity [5,6], or for 7 days in 4-week cycles at doses of 12 to 20 mg for 6 to 8 cycles [7], enable patients to receive a higher total chlorambucil dose (approximately 450 mg/m^2^) without a significant increase in the incidence and severity of side effects. The combination of more intensive chlorambucil schedules with rituximab results in response rates above 80% and median progression-free survival rates of two years or longer [7,8,9,10,11]. These outcomes seem superior to those achieved with chlorambucil pulses administered every 2 weeks in the CLL11 study, either as monotherapy or in combination with rituximab [3].

The combination of obinutuzumab and chlorambucil for front-line treatment of chronic lymphocytic leukemia became reimbursable in Croatia in 2017. Many of our centers continued using alternative chlorambucil schedules in combination with obinutuzumab. In order to examine the feasibility and efficacy of these combinations, we retrospectively collected data on the outcomes of patients receiving all types of chlorambucil and obinutuzumab combinations as front-line therapy for chronic lymphocytic leukemia in 2017 and 2018.

## 2. Materials and Methods

Patients were included in this analysis if they fulfilled the following entry criteria:

(1) Age > 18 years
(2) Chronic lymphocytic leukemia diagnosed according to standard criteria
(3) Previously untreated
(4) Started treatment with chlorambucil and obinutuzumab combinations (irrespective of the chlorambucil schedule and dose) in 2017 or 2018, outside of clinical studies
(5) Received at least one dose of each of the drugs.

Data on patient demographics (age, sex), baseline characteristics (Geriatric Cumulative Illness Rating Scale (CIRS-G) score [12,13], Binet stage, interphase fluorescence in situ hybridization [FISH]) and outcomes (overall survival, progression-free survival, side effects, modifications of planned treatment, cause of death) were retrieved from electronic hospital records. Chlorambucil schedules were divided into chlorambucil pulses (approximately 0.5 mg/kg every 2 weeks, two times per cycle, as in the CLL11 study), 7 days of chlorambucil per cycle (12 to 20 mg daily for 7 days every 4 weeks), and continuous high-dosed chlorambucil (10 to 20 mg daily until toxicity or response). Response to treatment was evaluated according to the relevant International Workshop Group on Chronic Lymphocytic Leukemia response criteria [14]. As is usual in routine clinical practice, bone marrow biopsies were frequently not repeated after the end of treatment to assess response; complete vs. partial remission were therefore defined by blood counts, and CT was used to determine lymph node and spleen size. Overall survival was defined as the period from the date of obinutuzumab or chlorambucil initiation (whichever occurred first) to death, irrespective of cause. Progression-free survival was defined as the period from the date of obinutuzumab or chlorambucil initiation (whichever occurred first) to treatment failure (including insufficient response and progression) or death. Prior to disease progression, the patients were followed up according to the standard practice of individual centers, usually every 3 months for the first 3 years after the end of treatment, and every 6 months thereafter.

Side effects were graded according to National Cancer Institute Common Terminology Criteria for Adverse Events grading system, version 5 [15].

Statistical analysis was conducted using freely available programs. Descriptive statistics, available within the MicroSoft Excel 2016^®^ program, were used to summarize baseline characteristics. Categorical variables were compared using Fisher’s exact [16] or G test [17], survival curves were plotted using the Kaplan–Meier method, and their difference was compared using the log-rank test [18].

The study was performed in accordance with all pertaining Croatian, European Union and international rules and regulations, and with the approval of the Ethical Committee of the University Hospital Centre Zagreb, Zagreb, Croatia, number 02/013AG. Since this was a retrospective study of anonymized patient data, a waiver of the requirement for informed consent was obtained.

## 3. Results

We identified 73 patients fulfilling the entry criteria (Table 1). The median age was 76 years and the range was 58 to 90 years; 41 (56%) were male and 32 (44%) were female. The median CIRS-G score was 9, and ranged from 2 to 30. Eleven patients had Binet disease stage A, 22 had Binet disease stage B, and 40 had Binet disease stage C. FISH was performed in 55 patients; a single one had del 17p, but in less than 10% of cells. Nineteen patients were treated with chlorambucil pulses administered every 2 weeks, 44 patients with cycles of 7 days of chlorambucil administered every 4 weeks, and 10 patients with continuous high-dosed chlorambucil. All patients received obinutuzumab as usual; first an application of 100 mg, followed by 900 mg if no serious infusion-related side effects occurred, followed by two applications of 1000 mg once weekly during the first cycle, and an additional five applications of 1000 mg every 4 weeks during the following five cycles of therapy. The total planned number of obinutuzumab cycles was six, and the total planned dose was 8000 mg. The premedication used before every obinutuzumab infusion included steroids, antihistaminergic drugs, and paracetamol. At the time of treatment start, tests for immunoglobulin heavy chain mutational status and *p53* sequencing were unavailable in Croatia.

Seven patients (10%) died during treatment: four of them due to infections, one due to Stevens–Johnson syndrome induced by chlorambucil, and two due to unrelated causes. Grade 4 granulocytopenia occurred in 15 patients (21%), trombocytopenia in 2 patients, and anemia in 1 patient. Infectious toxicity grade 3 to 5 occurred in 16 patients (22%) grade 3 hepatotoxicity in 2 patients, and chlorambucil-induced Stevens–Johnson syndrome grade 5 in 1 patient. Five patients (7%) had severe (grade 3 to 4) infusion reactions, causing obinutuzumab to be stopped in three patients (4%).

**Figure 1:**
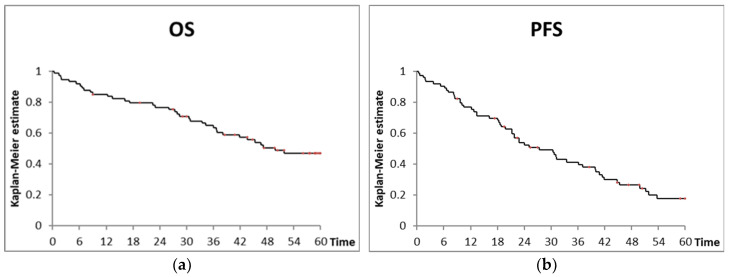
(**a**) Overall survival (OS) of the total cohort; (**b**) progression-free survival (PFS) of the total cohort.

The response rate was 89%, with 26 patients obtaining hematologic complete remission (CR), and 39 patients obtaining partial remission (PR). After a median follow up of the survivors of 59 months, 18 patients were alive without progression, an additional 17 patients were alive after progression, and 12 patients died after the end of treatment while in remission. The median progression-free survival time was 27 months, and the overall survival time was 50 months; the 2- and 5-year progression-free survival rates were 54% and 18%, and the overall survival rates were 77% and 47%, respectively (Figure 1). Sex, age, CIRS-G score, and FISH did not significantly influence progression-free survival and overall survival. Patients with Binet disease stages A and B had a trend towards superior progression-free survival (*p* = 0.096) and overall survival (*p* = 0.069) in comparison to those with Binet disease stage C.

Nineteen patients were treated with chlorambucil pulses administered every 2 weeks, two times per cycle. The median number of cycles of chlorambucil administered was six (range: 1 to 6), and of obinutuzumab, it was six (range: 1 to 6). Chlorambucil was administered as planned in 15 (79%) patients. One patient died during treatment. The cause of death was reported as being related neither to treatment, nor to chronic lymphocytic leukemia. Two patients had neutropenia gr. 4, one had thrombocytopenia gr. 4, and one had hepatotoxicity gr. 3.

Chlorambucil was administered in cycles of 7 days, every 4 weeks, in 44 patients. The median number of cycles of chlorambucil was five (range: 1 to 8), and of obinutuzumab, six (range: 1 to 6). Five patients died during treatment, three due to infections, one due to Stevens-Johnson syndrome and one due to unrelated causes. Ten had neutropenia gr. 4, one thrombocytopenia gr. 4, one anemia gr. 4, nine infections gr. 3–5, one hepatotoxicity gr. 3, and one Stevens-Johnson syndrome.

Ten patients were treated with continuous high dosed chlorambucil. The median duration of chlorambucil treatment was 4 weeks (range: 2 to 6). The median number of obinutuzumab cycles administered was six (range: 1 to 6). One patient died during treatment due to infection. Two had neutropenia gr. 4 and two had infections gr. 3–5. After starting obinutuzumab, chlorambucil had to be stopped or administered at low doses (less than 10 mg per day) due to neutropenia gr. 3 or higher in all the patients initially treated with high-dosed chlorambucil.

**Figure 2:**
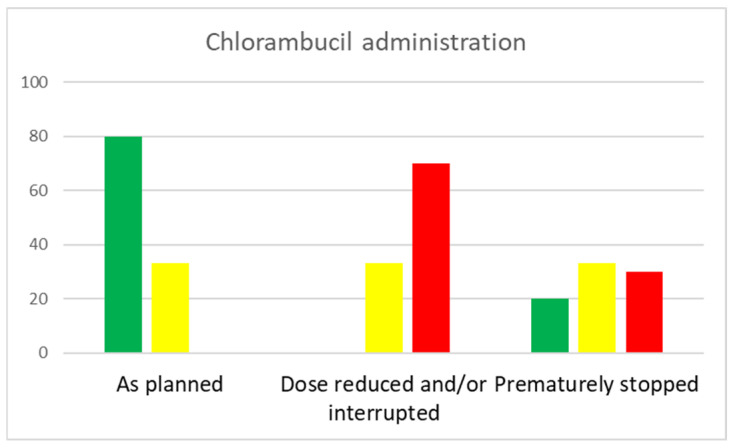
Feasibility of the combination of obinutuzumab with different chlorambucil schedules.

**Figure 3:**
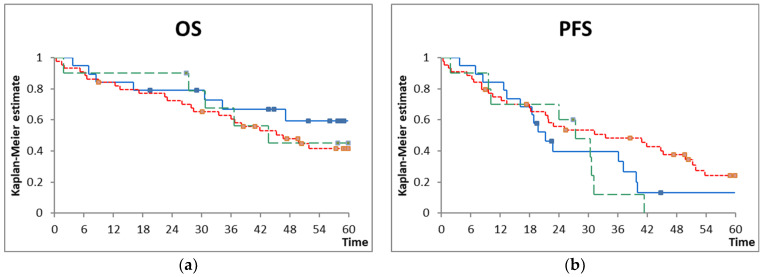
(**a**) Overall survival (OS) and (**b**) progression-free survival (PFS) according to chlorambucil schedule. Blue line = chlorambucil pulses every 2 weeks, two times per cycle; red line = cycles of 7 days of chlorambucil every 4 weeks; green line = continuous high-dosed chlorambucil (*p* = not significant for both outcomes).

Chlorambucil was administered as planned in 15 (79%) of the patients treated with chlorambucil pulses every 2 weeks two times per cycle, 15 (34%) of those treated with cycles of 7 days of chlorambucil administered every 4 weeks, and 0 of those treated with continuous high-dosed chlorambucil (*p* = 0.002) (Figure 2). Dose reductions, interruptions, and premature chlorambucil stopping were due to the side effects listed above, largely hematologic and infectious side effects. One patient treated with chlorambucil pulses every 2 weeks, five patients treated with cycles of 7 days of chlorambucil every 4 weeks, and one patient treated with continuous high-dosed chlorambucil died during treatment. The difference in treatment-related mortality between the groups was not statistically significant (*p* = 0.3293). All three combinations resulted in similar progression-free survival and overall survival (Figure 3).

Age and CIRS-G did not significantly affect premature stopping of chlorambucil, grade 3 to 4 side effects, and treatment-related mortality.

## 4. Discussion

This was a real life, non-interventional, retrospective analysis of patients starting treatment with chlorambucil and obinutuzumab combinations for chronic lymphocytic leukemia between 2017 and 2018. The cohort was most likely comprehensive, because all the Croatian hematology centers using obinutuzumab participated and, due to reimbursement rules, it was easy to identify all the treated patients, even retrospectively. However, as with any such cohort, treatment decisions might have been influenced by unrecognized factors, and conclusions on causality are therefore difficult to make. The decision on which chlorambucil schedule to use and when to adapt the planned treatment schedule was made by the treating physicians based on their personal and center preferences. There were no significant differences between the treatment groups regarding potential prognostic factors, like age, CIRS-G, and stage, but we cannot exclude the possibility that there were unrecognized biases influencing the outcomes.

In 2017 and 2018, Bruton tyrosine kinase inhibitors and venetoclax were not available for front-line therapy of chronic lymphocytic leukemia in Croatia, and the combination of obinutuzumab and chlorambucil was the treatment of choice for unfit and elderly patients [19]. The age (76 vs. 73 years) and median CIRS-G values (9 vs. 8) of our cohort were similar to those in the CLL11 study [3,4], but our series, as is usual in real-life studies, included patients that would not be eligible for the registration study, due to comorbidities and performance status (highest CIRS-G score: 30 vs. 20). Progression-free survival was remarkably similar (27 months in both series); overall survival was inferior in our cohort, possibly due to the inclusion of a number of patients that would not be eligible for the CLL11 study for the reasons mentioned before. Another reason for the difference in overall survival is the COVID-19 pandemic, which occurred during the follow up period of our study. It is well documented that, during the early years of the pandemic, this infection had a significant mortality rate in patients with chronic lymphocytic leukemia [20,21,22]. Toxicity was somewhat higher in our cohort than in the CLL11 study; the rate of grade 3 to 5 infections was 22% vs. 12%, and the rate of treatment-related mortality was 10% vs. 4%. This most probably reflects the differences in patient selection. An exception to the increased toxicity rate was a substantially lower grade 3 to 4 infusion reaction rate (5% vs. 20%). This can be explained by the widespread use of prolonged steroid premedication [23], or delaying obinutuzumab until the lymphocyte count had been reduced with chlorambucil [24]. Both methods have been shown to be effective in reducing the rates of severe infusion-related reactions, but were not used in the registration study. Treatment with obinutuzumab was feasible, and the vast majority of patients received the planned six cycles (eight applications, for a total dose of 8000 mg).

Besides the seminal CLL11 study, a number of real-life series of the combination of obinutuzumab and chlorambucil front-line therapy for chronic lymphocytic leukemia have been published by national groups and international consortia, such as ERIC (European Research Initiative on Chronic Lymphocytic Leukemia) [24,25,26,27,28,29,30]. All of them included elderly patients, with a median age of around 75 years and significant comorbidities (median CIRS-G score: 7 to 8). The response rates were between 76% and 95%. After a median follow up of less than 2 years, the median progression-free survival was between 28 and 33 months, with two outliers. In one of these studies, the median progression-free survival was 49 months, but with a very large confidence interval (12 to 80 months), due to an unusual flattening of the survival curve immediately above 50%, which would suggest that this is probably a statistical artifact [26]. In the second study, the response rate was 95%, no grade 4 to 5 toxicities occurred, and after a median follow up of 18 months, the progression-free survival rate at 30 months was 62% [30]. The reason for this discrepancy is unclear; patient selection might be a possible explanation. While our results are at the lower end of the spectrum, they are more mature (median follow up of survivors of almost 5 years), patients’ baseline characteristics were inferior (median age: 76 years, and CIRS-G score: 9), and the COVID-19 pandemic, which had a substantial effect on patients with lymphoid malignancies, including chronic lymphocytic leukemia, occurred during the follow up.

To our knowledge, this is the only published study of the combination of obinutuzumab and chlorambucil in front-line therapy of chronic lymphocytic leukemia comparing different chlorambucil schedules. Alternative chlorambucil schedules did not improve outcomes in comparison to the one used in the registration study. This was probably a consequence of the fact that the treatment with more intensive chlorambucil schedules had to be modified more frequently, the dose of chlorambucil reduced, or the treatment stopped prematurely, due to more frequent side effects and greatly increased hematologic and infectious toxicity. It was therefore not possible to administer the planned higher total dose of chlorambucil. Especially unfeasible was the combination of obinutuzumab with continuous high-dosed chlorambucil, which could not be administered as planned in any patient. This finding is another indicator of the well-known increased hematotoxicity of obinutuzumab in comparison to rituximab.

## 5. Conclusions

The real-life outcomes of the combination of obinutuzumab with chlorambucil in front-line therapy for chronic lymphocytic leukemia were very similar to those reported in the registration study. It seems that it is possible to reduce the risk of serious infusion-related side effects by prolonged steroid premedication or lymphocyte count reduction with chlorambucil monotherapy. Alternative chlorambucil schedules, designed to enable the administration of higher total doses of the drug, are not well tolerated, and do not improve outcomes. However, nowadays, the place of the combination of obinutuzumab and chlorambucil in front-line treatment of chronic lymphocytic leukemia is significantly diminished, because the results are generally inferior to those achieved with new targeted agents, the Bruton tyrosine kinase inhibitors ibrutinib, acalabrutinib, and Zanubrutinib, and the BCL2 inhibitor venetoclax, which achieve progression-free survival rates of over 80% at 2 years [31,32,33,34].

## Figures and Tables

**Table 1:** Patient characteristics.

Characteristic	
Age (median/range)	76 years/58–90 years
Sex (male/female)	41/32
Binet stage (A/B/C)	11/22/40
CIRS-G (median/range)	9/2–30
FISH (not performed/normal/del13/+12/del11/del17p)	18/16/16/12/10/1
Chlorambucil schedule * (pulse/7 d/cont)	19/44/10

* pulse = 0.5 mg/kg, 2 wks; 7 d = 12 to 20 mg daily for 7 days, 4 wks; cont = 10 to 20 mg daily, continuous until response or toxicity.

## Data Availability

The data presented in this study are available on request from the corresponding author. Due to the informed consent waiver, any requests for the original data should be sent to the corresponding author, and must be approved by a Croatian Ethics Committee.
